# DgbZIP3 interacts with DgbZIP2 to increase the expression of *DgPOD* for cold stress tolerance in chrysanthemum

**DOI:** 10.1093/hr/uhac105

**Published:** 2022-05-17

**Authors:** Huiru Bai, Xiaoqin Liao, Xin Li, Bei Wang, Yunchen Luo, Xiaohan Yang, Yuchen Tian, Lei Zhang, Fan Zhang, Yuanzhi Pan, Beibei Jiang, Yin Jia, Qinglin Liu

**Affiliations:** Department of Ornamental Horticulture, Sichuan Agricultural University, 211 Huimin Road, Wenjiang District, Chengdu, Sichuan 611130, China; Department of Ornamental Horticulture, Sichuan Agricultural University, 211 Huimin Road, Wenjiang District, Chengdu, Sichuan 611130, China; Department of Ornamental Horticulture, Sichuan Agricultural University, 211 Huimin Road, Wenjiang District, Chengdu, Sichuan 611130, China; Department of Ornamental Horticulture, Sichuan Agricultural University, 211 Huimin Road, Wenjiang District, Chengdu, Sichuan 611130, China; Department of Ornamental Horticulture, Sichuan Agricultural University, 211 Huimin Road, Wenjiang District, Chengdu, Sichuan 611130, China; Department of Ornamental Horticulture, Sichuan Agricultural University, 211 Huimin Road, Wenjiang District, Chengdu, Sichuan 611130, China; Department of Ornamental Horticulture, Sichuan Agricultural University, 211 Huimin Road, Wenjiang District, Chengdu, Sichuan 611130, China; Department of Ornamental Horticulture, Sichuan Agricultural University, 211 Huimin Road, Wenjiang District, Chengdu, Sichuan 611130, China; Department of Ornamental Horticulture, Sichuan Agricultural University, 211 Huimin Road, Wenjiang District, Chengdu, Sichuan 611130, China; Department of Ornamental Horticulture, Sichuan Agricultural University, 211 Huimin Road, Wenjiang District, Chengdu, Sichuan 611130, China; Department of Ornamental Horticulture, Sichuan Agricultural University, 211 Huimin Road, Wenjiang District, Chengdu, Sichuan 611130, China; Department of Ornamental Horticulture, Sichuan Agricultural University, 211 Huimin Road, Wenjiang District, Chengdu, Sichuan 611130, China; Department of Ornamental Horticulture, Sichuan Agricultural University, 211 Huimin Road, Wenjiang District, Chengdu, Sichuan 611130, China

## Abstract

The bZIP transcription factor plays a very important role in abiotic stresses, e.g. drought, salt, and low-temperature stress, but the mechanism of action at low temperature is still unclear. In this study, overexpression of *DgbZIP3* led to increased tolerance of chrysanthemum (*Chrysanthemum morifolium* Ramat.) to cold stress, whereas antisense suppression of *DgbZIP3* resulted in decreased tolerance. Electrophoretic mobility shift assay (EMSA), chromatin immunoprecipitation (ChIP), luciferase complementary imaging analysis (LCI), and dual-luciferase reporter gene detection (DLA) experiments indicated that DgbZIP3 directly bound to the promoter of *DgPOD* and activated its expression. DgbZIP2 was identified as a DgbZIP3-interacting protein using yeast two-hybrid, co-immunoprecipitation, LCI, and bimolecular fluorescence complementation assays. Overexpression of *DgbZIP2* led to increased tolerance of chrysanthemum to cold stress, whereas antisense suppression of *DgbZIP2* resulted in decreased tolerance. A ChIP–qPCR experiment showed that DgbZIP2 was highly enriched in the promoter of *DgPOD*, while DLA, EMSA, and LCI experiments further showed that DgbZIP2 could not directly regulate the expression of *DgPOD*. The above results show that DgbZIP3 interacts with DgbZIP2 to regulate the expression of *DgPOD* to promote an increase in peroxidase activity, thereby regulating the balance of reactive oxygen species and improving the tolerance of chrysanthemum to low-temperature stress.

## Introduction

Low temperature, as the main abiotic stress, reduces the yield and quality of plants and limits the regional distribution of plants during plant growth and development [1]. Plants will effect changes in the expression of related genes under low-temperature stress, promoting a series of physiological, biochemical, and molecular reactions [2, 3].

To cope with abiotic stress, plants regulate the expression of a series of genes related to this type of stress through internal signaling pathways by the interaction between transcription factors and *cis* elements encoded in the promoter region of abiotic stress-related genes, so as to improve plant stress resistance. Most transcription factors take an important role in plant abiotic stress, such as bZIP, NAC, WRKY, MADS, AP2/EREBP, MYB, bHLH, zinc finger protein, and MYC [4, 5].

Most bZIP transcription factors are composed of a DNA-binding domain composed of 18 basic amino acid residues (N-X7-R/K) at the N-terminus [6] and 2 basic domains in the leucine zipper region at the C-terminus that are involved in oligomerization [7]. bZIP transcription factors take an important part in abiotic stresses, e.g. low-temperature, salt, and drought stresses, in plants [8].

bZIP can inhibit or activate the expression of multiple downstream genes by interacting with the *cis*-acting elements G-box, A-box, ABRE, LTRE, and C-box in the promoter region, thereby participating in the process of transcriptional regulation. In response to abiotic stress, *Glycine max GmbZIP44*, *GmbZIP62*, and *GmbZIP78* enhance resistance to salt stress and cold stress in transgenic *Arabidopsis* [9], and overexpression of *ThbZIP1* in *Setaria striata* can significantly increase the tolerance of *Arabidopsis* to drought and salt stress [10]. Overexpression of *OsbZIP71* enhanced the resistance of rice to drought and salt stress. On the contrary, the knockout of *OsbZIP71* made rice more sensitive to drought, abscisic acid, and salt stress [11]. The expression level of *AtbZIP1* was significantly increased in *Arabidopsis* under cold stress, salinity, and drought stress; overexpression of *AtbZIP1* increased the tolerance of *Arabidopsis* to drought and salt stress [12].

Low temperature will affect the shape of flowers, cause the rosette phenomenon in plants, and greatly reduce the economic benefits and ornamental value of chrysanthemum; it is one of the most important abiotic stresses on chrysanthemum [13]. The constitutive expression of *CdICE1* in chrysanthemum improved low-temperature tolerance [14]. Overexpression of *DgTIL1* improved the tolerance of chrysanthemum to low-temperature stress, and the lysine crotonylation of DgTIL1 prevented the degradation of the non-specific lipid transfer protein DgnsLTP in chrysanthemum, thereby promoting the activity of peroxidase (POD) and reactive oxygen species (ROS) scavenging and therefore improving the tolerance of chrysanthemum to low-temperature stress [15]. DgMYB2 promotes increased glutathione peroxidase (GPX) activity by regulating the expression level of DgGPX1, reduces the accumulation of ROS in chrysanthemum, and improves the tolerance of chrysanthemum to low-temperature stress [16]. Decrotonylation of DgGPX1 at lysine K220 can promote the enhancement of GPX activity, thereby reducing the accumulation of ROS in chrysanthemum and improving cold resistance [17]. Overexpression of *DgC3H1* in chrysanthemum enhances cold tolerance by modulating the plant’s ROS system [18]. However, bZIP transcription factors are less studied in chrysanthemum.

In our research, we found that *DgbZIP3* overexpression increases cold tolerance in chrysanthemum. In addition, antisense inhibition reduces the tolerance of chrysanthemum to cold stress. Electrophoretic mobility shift assay (EMSA), chromatin immunoprecipitation (ChIP), luciferase complementary imaging (LCI), and dual-luciferase reporter gene detection assay (DLA) analyses showed that DgbZIP3 can be combined with the *DgPOD* promoter to initiate the expression of *DgPOD* and improve the ability of chrysanthemum to resist low-temperature stress. Yeast two-hybrid (Y2H), bimolecular fluorescence complementation assay (BIFC), LCI, and co-immunoprecipitation (Co-IP) analyses indicated that DgbZIP3 interacts with DgbZIP2. DgbZIP2 cannot directly regulate the expression of *DgPOD*; after interacting with DgbZIP3, it can increase the expression of *DgPOD*, enhance the activity of POD, and regulate the balance of ROS, thereby improving the cold resistance of chrysanthemum.

## Results

### *DgbZIP3* is responsive to low temperature

Transcriptome analysis (accession number GSE117262) of chrysanthemum seedlings was performed under normal treatment (at 25°C) and cold treatment (24 hours at 4°C and 4 hours at −4°C). The analysis results indicated that the transcription level of *DgbZIP3* (GenBank accession number MW528211) in chrysanthemum was significantly increased after low-temperature treatment. *DgbZIP3* has an open reading frame (ORF) with a length of 1008 bp, encoding a polypeptide of 336 amino acids (Supplementary Data Fig. S1). Multiple comparisons of the amino acid sequence of *DgbZIP3* with other homologous amino acid sequences indicated that DgbZIP3 contains a conserved domain, NRESARRSR (N-X7-R/K) (Supplementary Data Fig. S2a). Phylogenetic analysis showed that DgbZIP3 belongs to the bZIP transcription factor family and is closely related to TcbZIP9 in *Tanacetum cinerariifolium* (Supplementary Data Fig. S2b).

We determined the transcriptional abundance of *DgbZIP3* in different tissues of wild-type (WT) chrysanthemum seedlings by qRT–PCR and found that the transcription level of *DgbZIP3* in chrysanthemum leaves was significantly higher than that in stems and roots (Supplementary Data Fig. S3a). We subjected chrysanthemum to cold stress for 3 hours; the transcript level of *DgbZIP3* in leaves started to increase gradually and reached a peak after 12 hours (Supplementary Data Fig. S3b).

### DgbZIP3 interacts with DgbZIP2

**Figure 1:**
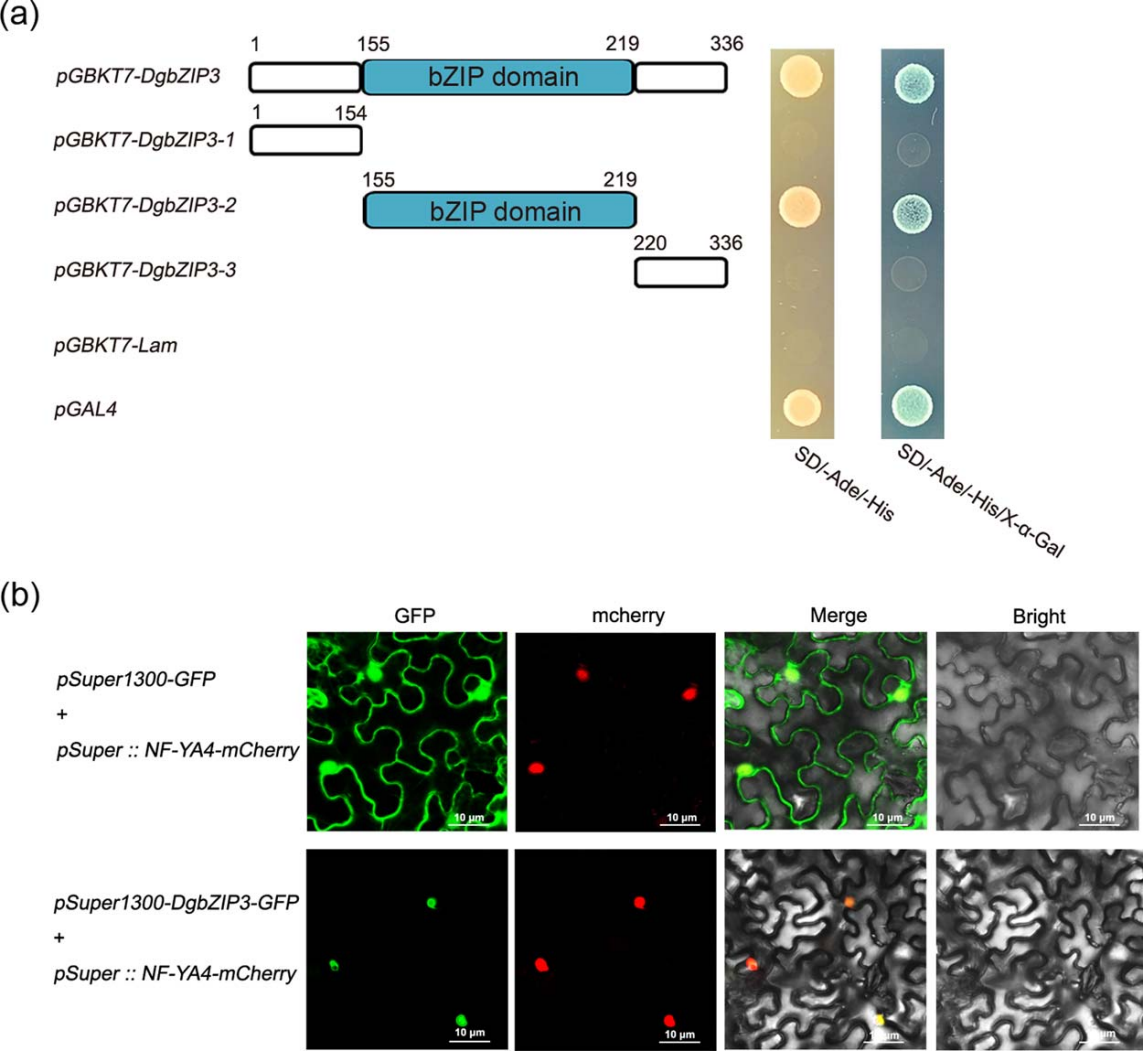
Y1H analysis and subcellular localization*.*  **a** Y1H analysis of DgbZIP3. **b** Subcellular localization of DgbZIP3. Scale bars = 10 μm.

In order to clarify the transcriptional activation region of DgbZIP3, we constructed three deletion fragments of DgbZIP3. The yeast vector *pGBKT7* was ligated with three fragments, and its activation activity was analyzed by yeast single hybridization (Y1H). The experimental results showed that *pGBKT7-DgbZIP3*, *pGBKT7-DgbZIP3-2*, and *pGAL4* grew normally on SD/−Ade/−His medium and were blue on medium supplemented with X-α-Gal (Fig. 1a), which has transcriptional activation activity. This indicated that the polypeptide region 155–219 belongs to the transcriptional activation region of DgbZIP3.

Co-expression of *pSuper::NF-YA4-mCherry* and *pSuper1300-DgbZIP3-GFP* in tobacco leaves was performed to analyze the subcellular localization of DgbZIP3, and the results indicated that DgbZIP3 was localized in the nucleus (Fig. 1b).

To further identify potential DgbZIP3 interaction factors, we used chrysanthemum *pGBKT7-DgbZIP3* bait vector and empty *pGADT7* to transform the Y2HGold yeast strain for Y2H screening and identified a potential interacting protein, bZIP2, which was named *DgbZIP2* (GenBank accession number MW528212).

**Figure 2:**
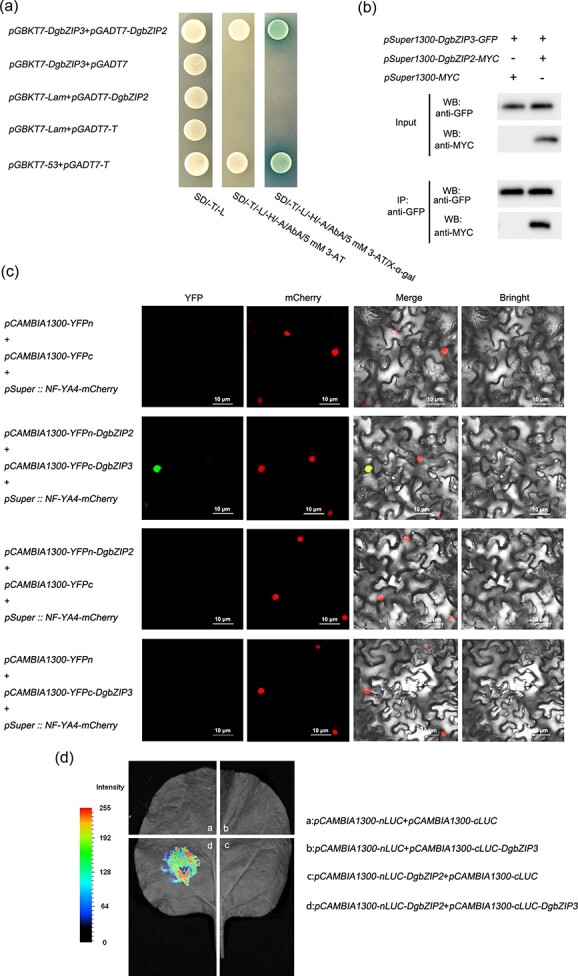
DgbZIP3 interacts with DgbZIP2. **a** Y2H verifies the interaction between DgbZIP3 and DgbZIP2. **b** Co-IP assay. Co-expression of *pSuper1300-DgbZIP3-GFP* and *pSuper1300-MYC* was used as a control. **c** BIFC demonstrated that DgbZIP3 interacts with DgbZIP2 in tobacco. Scale bars = 10 μM. **d** DgbZIP3 and DgbZIP2 LCI analysis.

Y2H, Co-IP, BIFC, and LCI experiments (Fig. 2a–d) further confirmed the interaction between DgbZIP3 and DgbZIP2.

The ORF length of *DgbZIP2* is 1170 bp, which encodes a 140-amino acid protein (Supplementary Data Fig. S4). Multiple comparisons between the amino acid sequence of *DgbZIP2* and other homologous amino acid sequences in DNAMAN indicated that DgbZIP2 contains a conserved domain, NRESARRSR (N-X7-R/K) (Supplementary Data Fig. S5a). Phylogenetic analysis showed that DgbZIP2 belongs to the bZIP transcription factor family and is closely related to TcbZIP53 in *T. cinerariifolium* (Supplementary Data Fig. S5b).

Co-expression of *pSuper::NF-YA4-mCherry* and *pSuper1300-DgbZIP2-GFP* in tobacco leaves was performed to analyze the subcellular localization of DgbZIP2, and the results indicated that DgbZIP2 was localized in the nucleus (Supplementary Data Fig. S6).

### *DgbZIP2* is responsive to low temperature

We detected the transcriptional abundance of *DgbZIP2* in different tissues of WT chrysanthemum seedlings by qRT–PCR, and found that the transcription level of *DgbZIP2* in chrysanthemum leaves was significantly higher than that in stems and roots (Supplementary Data Fig. S7a). We subjected chrysanthemum to cold stress for 3 hours; the transcript level of *DgbZIP2* in leaves started to increase gradually and reached a peak after 12 hours (Supplementary Data Fig. S7b).

### Overexpression of *DgbZIP3* led to increased tolerance of chrysanthemum to cold stress, whereas antisense suppression of *DgbZIP3* resulted in decreased tolerance

**Figure 3:**
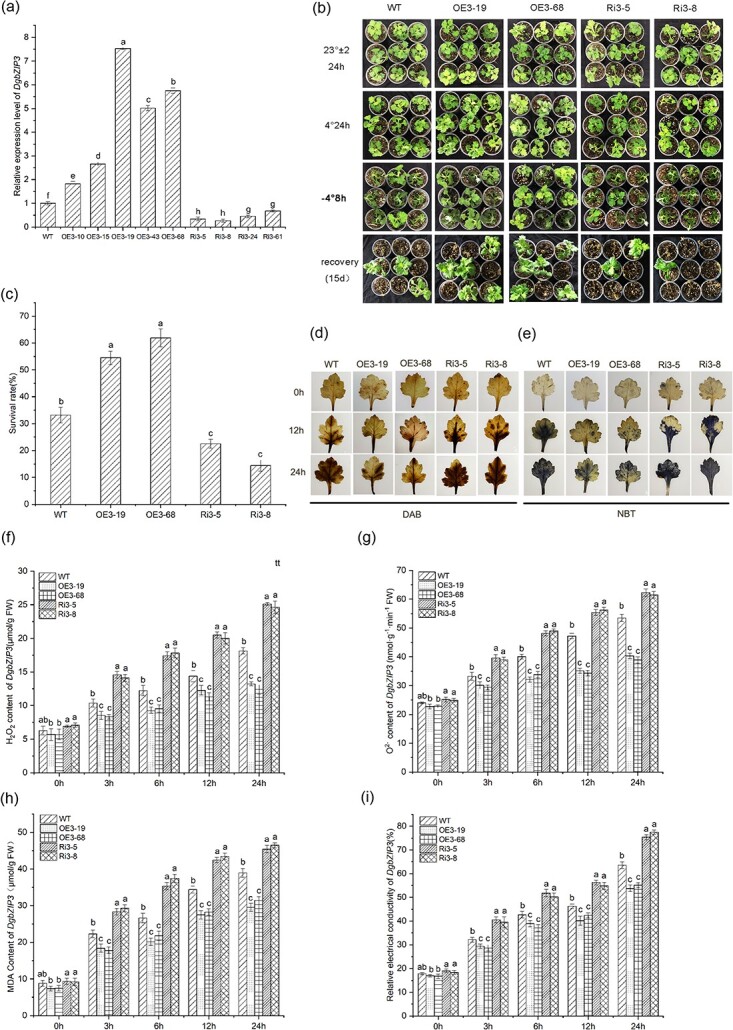
*DgbZIP3* overexpressed fewer ROS products in chrysanthemum. **a** Transcript levels of *DgbZIP3* in transgenic and WT lines. **b** Phenotypic changes after cold stress in transgenic and WT lines. **c** Survival of WT and *DgbZIP2* transgenic lines after 15 days of recovery under 25°C day/22°C night control. **d**, **e** DAB (**d**) and NBT (**e**) histochemical staining. **f**, **g** Determination of H_2_O_2_ (**f**) and O^2−^ (**g**) accumulation in chrysanthemum. **h**, **i** Analysis of MDA (**h**) and relative electrolyte leakage (**i**).

To verify the role of *DgbZIP3* in cold stress, *Agrobacterium*-mediated transformation of WT chrysanthemum was used to obtain transgenic plants. The transcript abundance of *DgbZIP3* in nine transgenic lines was determined by qRT–PCR (Fig. 3a). We used the OE3-19 and OE3-68 lines with significantly increased transcript levels and the Ri3-5 and Ri3-8 lines with significantly reduced transcript levels to conduct cold stress experiments. With increasing low-temperature treatment time and decreasing temperature, compared with WT, the Ri3-5 and Ri3-8 lines had the highest degree of wilting, lodging, and dehydration. The OE3-19 and OE3-68 lines had the lowest degree of wilting, lodging, and dehydration (Fig. 3b). The survival rate of WT was 33.18%, the survival rates of Ri3-5 and Ri3-8 were 22.52 and 14.37%, and the survival rates of OE3-52 and OE3-138 were 54.49 and 61.89%, respectively, after 2 weeks of recovery from low temperature (Fig. 3c).

Diaminobenzidine (DAB) and nitroblue tetrazolium (NBT) histochemical staining methods were used to stain the leaves of WT and transgenic chrysanthemum (Fig. 3d and e). The results showed that with increasing cold stress treatment time there were more spots on the leaves and leaf color was darker. Compared with WT, the Ri3 line had the most blue or brown spots and the most serious oxidative damage, while the OE3 line had the least blue or brown spots and the least oxidative damage. Quantitative analysis of H_2_O_2_ and the O^2−^ content showed the same results (Fig. 3f and g). In addition, the malondialdehyde (MDA) content and relative electrolyte permeability (REC) level increased gradually with increasing cold stress treatment time. After 24 hours, the MDA content and relative electrolyte permeability in the Ri3 line were significantly higher than in WT, while the MDA content and REC in the OE3 line were lower than in WT (Fig. 3h and i), indicating the greatest membrane damage in the antisense inhibition line, followed by WT and the overexpression line. These results indicated that, under cold stress, compared with WT, the *DgbZIP3* overexpression line had less ROS accumulation, while the antisense suppressor line had more ROS accumulation, which shows that the overexpression of *DgbZIP3* in chrysanthemum can improve cold resistance.

In order to analyze the mechanism of ROS scavenging in chrysanthemum, the activities of POD, catalase (CAT), superoxide dismutase (SOD), and anti-ascorbate peroxidase (APX) in the WT, overexpression and antisense suppression lines under normal conditions and cold stress were determined. The results indicated that although SOD, CAT, and APX activities increased gradually with the extension of the low-temperature treatment time, the difference between WT, overexpression, and antisense suppressor lines was not significant (Supplementary Data Fig. S8a–c). Compared with WT, POD activity was highest in the overexpressing line, while POD activity was lowest in the antisense suppressor line (Supplementary Data Fig. S8d). This shows that the overexpression of *DgbZIP3* increased the activity of POD, reduced the accumulation of ROS, and enhanced cold resistance, while antisense inhibition reduced the activity of POD and increased the accumulation of ROS, resulting in weaker cold resistance.

### Overexpression of *DgbZIP2* leads to increased tolerance of chrysanthemum to cold stress, whereas antisense suppression of *DgbZIP2* resulted in decreased tolerance

**Figure 4:**
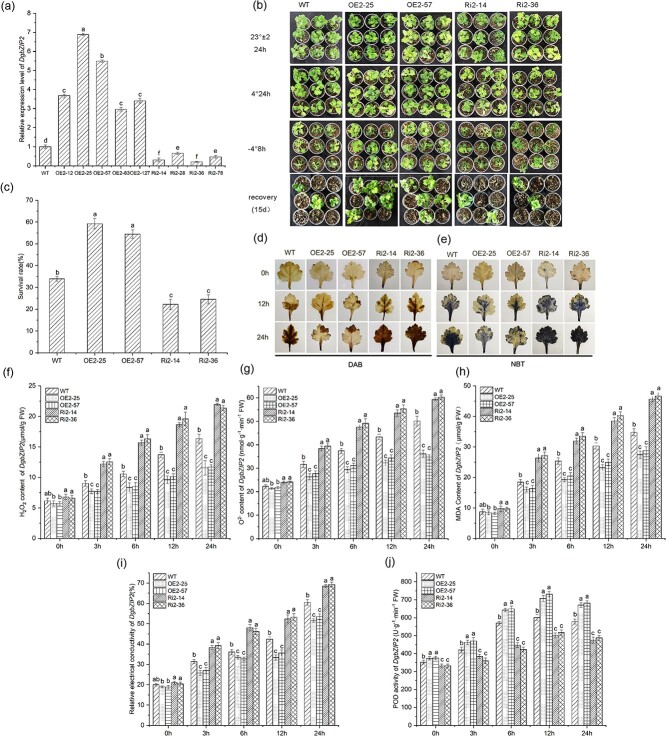
*DgbZIP2* overexpressed fewer ROS products in chrysanthemum. **a** Transcript levels of *DgbZIP2* in transgenic and WT lines. **b** Phenotypic changes after cold stress in transgenic and WT lines. **c** Survival of WT and *DgbZIP2* transgenic lines after 15 days of recovery under 25°C day/22°C night control. **d**, **e** DAB (**d**) and NBT (**e**) histochemical staining. **f**, **g** H_2_O_2_ (**f**) and O^2−^ (**g**) accumulation in chrysanthemum. **h**, **i** Analysis of MDA (**h**) and relative electrolyte leakage (**i**). **j** POD activity under low temperature.

The transcription level of *DgbZIP2* was determined in nine transgenic lines by qRT–PCR. Under low-temperature stress, the OE2-25 and OE2-57 lines had the highest expression levels, followed by WT, while the Ri2-14 and Ri2-36 lines had the lowest expression levels (Fig. 4a). The degree of wilting, lodging, and dehydration of the Ri2 line was the most serious under low-temperature stress, followed by WT and the OE2 line (Fig. 4b). After 2 weeks of recovery from cold stress, the WT survival rate was 33.96%, the survival rates of Ri2-14 and Ri2-36 were 22.19 and 24.52%, and the survival rates of OE2-25 and OE2-57 were 59.26 and 54.47%, respectively (Fig. 4c).

DAB and NBT histochemical staining methods (Fig. 4d and e) were used to stain the leaves of the WT, overexpression, and antisense suppressor lines of chrysanthemum to analyze the accumulation of H_2_O_2_ and O^2−^ under cold stress. The results indicated that, compared with the WT line, the Ri2 line had the most blue or brown spots and the most severe oxidation, while the OE2 line had the fewest blue or brown spots and the least oxidative damage. Quantitative analysis of H_2_O_2_ and O^2−^ showed the same results (Fig. 4f and g). In addition, the content of MDA and REC level gradually increased with the extension of the cold stress time. After 24 hours, the MDA content and REC level in the Ri2 line were the highest, followed by WT, and the MDA content and REC level in OE2 line were the lowest, indicating that the cell membrane damage of the antisense inhibitor line was the greatest, followed by the overexpression and WT lines (Fig. 4h and i). The above experimental results showed that under low-temperature stress the accumulation of ROS was lower in the *DgbZIP2* overexpression lines, while ROS accumulation was higher in the antisense suppressor lines, which showed that *DgbZIP2* overexpression can increase the cold resistance of chrysanthemum.

In order to explore the mechanism of ROS clearance, we measured POD activity in WT and transgenic lines under normal conditions and cold stress. The results indicated that the activity of POD was highest in the overexpression line, followed by WT, and lowest in the antisense suppression line (Fig. 4j). This shows that overexpression of *DgbZIP2* increased the activity of POD, reduced the accumulation of ROS, and improved cold resistance, while the antisense strain reduced the activity of POD, increased the accumulation of ROS, and reduced cold resistance.

### DgbZIP2 and DgbZIP3 cooperatively promote *DgPOD* expression

**Figure 5:**
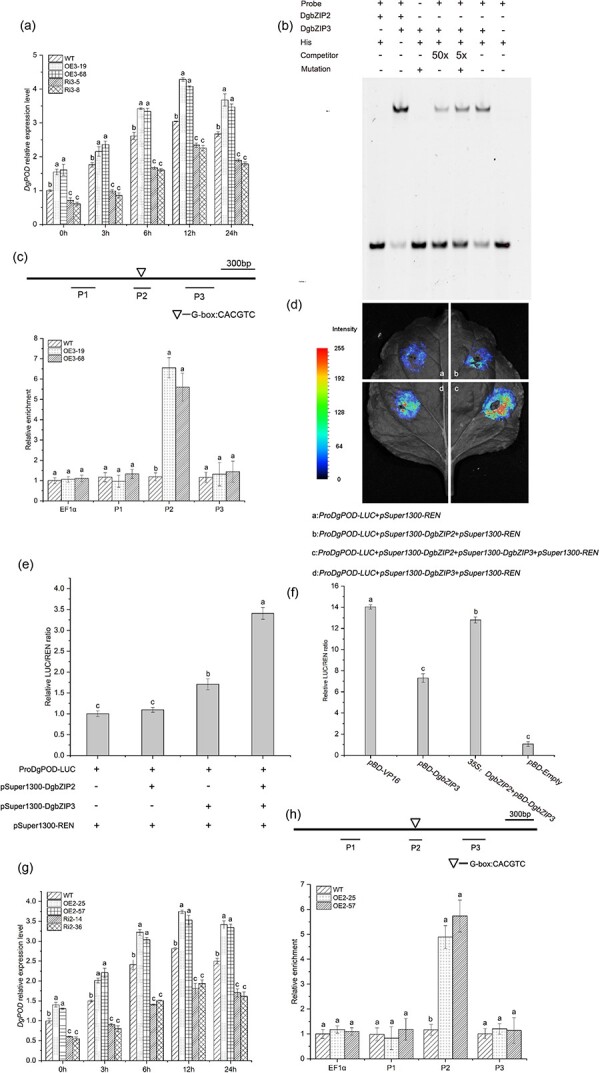
DgbZIP3 and DgbZIP2 bind to the promoter of *DgPOD*. **a** Expression level of *DgPOD* in WT and *DgbZIP3* transgenic lines. **b** EMSA assay. Left to right: DgbZIP2 with 6-FAM-labeled probe and His protein; DgbZIP2 and DgbZIP3 with 6-FAM-labeled probe; DgbZIP3 with 6-FAM-labeled mutant probe and His protein; DgbZIP3 with 6-FAM-labeled probe and 50× unlabeled probe and His protein; DgbZIP3 with 6-FAM-labeled probe and 5× unlabeled probe and His protein; DgbZIP3 with 6-FAM-labeled probe and His protein; and 6-FAM-labeled probe and His protein. **c** ChIP–PCR assay for DgbZIP3. G-box, CACGTC components (CORE) in the *DgPOD* promoter; P1–P3, various segments of the promoter sequence, among which P2 contains the CORE element. **d** LCI analysis. **e** DLA analysis. **f** Transcriptional activation activity. **g** Expression level of *DgPOD* in WT and *DgbZIP2* transgenic lines. **h** ChIP–PCR assay for DgbZIP2.

Under low-temperature treatment, the expression level of *DgPOD* in the *DgbZIP3* overexpression line was significantly higher than that of WT, while the expression level in the antisense suppression line was lower than that of WT (Fig. 5a).

In order to understand the direct relationship between DgbZIP3 and the *DgPOD* promoter, the *DgPOD* promoter was cloned. According to the specificity of binding to DNA, a plant’s bZIP protein binds to the G-box element (CACGTG) [19], and the promoter of *DgPOD* contains the G-box element (CACGTG). EMSA analysis showed that DgbZIP3 was able to bind to the *DgPOD* promoter (Fig. 5b). This result was confirmed by the ChIP–qPCR (Fig. 5c), LCI experiments (Fig. 5d), and DLA detection (Fig. 5e and f). These results indicated that there is a protein–DNA interaction between DgbZIP3 and the *cis*-acting element G-box (CACGTG) in the *DgPOD* promoter region, indicating that DgbZIP3 can directly regulate the expression of *DgPOD* and improve the cold resistance of plants.

In order to illustrate the ability of *DgbZIP2* and *DgPOD* to regulate low-temperature stress, we used qRT–PCR to detect the transcript level of *DgPOD* in the WT, overexpression, and antisense suppressor lines. The results were consistent with the results of POD activity; the OE2 line had the highest transcript level and the Ri2 line had the lowest transcript level compared with WT (Fig. 5g). The ChIP–qPCR experiment (Fig. 5h) showed that DgbZIP2 was highly enriched in the P2 region of the G-box (CACGTG) on the *DgPOD* promoter. EMSA analysis further showed that DgbZIP2 could not directly bind to the *DgPOD* promoter, but DgbZIP2 interacted DgbZIP3 and then bound to the *DgPOD* promoter to enhance the expression of *DgPOD* (Fig. 5b). This conclusion is supported by the LCI experiments (Fig. 5d) and DLA detection (Fig. 5e).

## Discussion

At present, the bZIP transcription factor has been studied in many plants [7, 20–27] and the bZIP transcription factor family has been determined to respond to abiotic stresses such as drought, salt, and low temperature. Overexpression of *OsbZIP52* enhances tolerance to cold stress and drought in rice [28]. Low temperature, mechanical damage, and salt stress all induce the expression of *TabZIP1* in wheat [29]. Overexpression of *Wlip19* improves tobacco tolerance to cold stress [30]. Under cold stress, *Arabidopsis* plants overexpressing *CsbZIP6* are more resistant to freezing than WT [31]. Overexpression of wheat *TabZIP14-B* and *TabZIP60* in *Arabidopsis* enhanced the tolerance of *Arabidopsis* to low temperatures and salt stress [32, 33]. We isolated a bZIP transcription factor gene from chrysanthemum and named it *DgbZIP3*. Sequence analysis and phylogenetic analysis showed that the protein has a conserved NRESARRSR domain (Supplementary Data Fig. S2a), which has close homology with TcbZIP9 in *T. cinerariifolium* (Supplementary Data Fig. S2b). Y1H analysis (Fig. 1a) showed that DgbZIP3 has transcriptional activation activity. Subcellular localization analysis further showed that DgbZIP3 is located in the nucleus and is a member of the bZIP transcription factor family (Fig. 1b).

Plant resistance to abiotic stress can be measured by MDA content and REC level [34, 35]. Under low-temperature stress, the level of REC and MDA content continued to increase with the extension of the stress time (Fig. 3h and i), and the contents of H_2_O_2_ and O^2−^ also gradually increased (Fig. 3f and g). ROS gradually accumulated in chrysanthemum under cold stress. Excessive accumulation of ROS in plants will destroy the integrity of cell membranes, and cause toxins to accumulate and lead to death [36]. Damage of plant cell membranes can be measured by relative electrolyte permeability level and MDA content [37]. Under cold stress, compared with WT, REC, and MDA in the *Arabidopsis CsbZIP6* overexpression line increased, and the tolerance to low-temperature stress was reduced, showing that *CsbZIP6* was a negative regulator of cold stress [31]. The experimental results showed that, compared with the WT line, the REC level, MDA content, and ROS accumulation (such as H_2_O_2_ and O^2−^) in the antisense suppressor line were significantly higher, while the REC level and MDA content were lower in the overexpression line, and ROS accumulation was minimal (such as H_2_O_2_ and O^2−^). This showed that *DgbZIP3* can improve the stability of the cell membrane under cold stress. In order to reduce oxidative damage under low-temperature stress, plants reduce or eliminate excessive ROS accumulation by regulating the activity of antioxidant enzymes so that the ROS system in the plant is in a balanced state [38]. POD acts as an important antioxidant enzyme, eliminating ROS toxicity and maintaining ROS homeostasis [39, 40]. In our study, under cold stress, POD activity Supplementary Data Fig. S8d) and the relative expression level of *DgPOD* (Fig. 5a) in the *DgbZIP3* overexpression lines were significantly higher than in WT, while POD activity and the relative expression level of *DgPOD* in the antisense suppressor lines were significantly lower than in WT. This shows that *DgbZIP3* overexpression in chrysanthemum can decrease or eliminate ROS toxicity, strengthen the protection of cell membrane integrity, and reduce oxidative stress so as to improve the ability of chrysanthemum to resist low-temperature stress.

bZIP73*^Jap^* binds to the promoter region of POD precursor genes in *bZIP73^Jap^* overexpression seedlings, upregulates their expression, and improves the cold resistance of rice [41]. TaAREB3 can bind to the promoters of *COR47*, *COR15A*, *RD29A*, and *RD29B* to initiate activity, increasing tolerance to low temperature and drought in *Arabidopsis* [42]. OsbZIP52 can specifically bind to the *cis*-acting element G-box on the downstream gene promoter and initiate the expression of downstream *OsTPP1*, *OsLEA3*, *Rab25*, and other abiotic stress-related genes, resulting in the overexpression of *OsbZIP52* reducing the tolerance of rice to low-temperature stress [28]. The bZIP protein mlip15 binds to the promoter region of the *Adh1* and improved the tolerance of maize to low-temperature stress [43]. Overexpression of *MdHY5* improves the ability of *Arabidopsis* to resist low-temperature stress. Transient expression analysis and EMSA analysis showed that MdHY5 can bind to the G-box on the *MdCBF1* promoter, promote the expression of the *COR*, and improve cold resistance [44]. Our analysis by EMSA, DLA, LCI, and ChIP–qPCR (Fig. 5b–f) showed that DgbZIP3 could combine with the *cis*-acting element G-box (CACGTC) in the *DgPOD* promoter region to promote the expression of *DgPOD*, regulate the balance of ROS, and enhance the resistance of chrysanthemum to low-temperature stress.

To further analyze the mechanism by which DgbZIP3 regulates cold stress, we screened out the DgbZIP3 interaction with DgbZIP2. We further verified the interaction of DgbZIP3 and DgbZIP2 in the nucleus through BIFC, LCI, Y2H, and Co-IP experiments (Fig. 2a–d). We isolated and identified *DgbZIP2* from chrysanthemum. Subcellular localization analysis indicated that DgbZIP2 is located in the nucleus, which belongs to the bZIP transcription factor family (Supplementary Data Fig. S6). Under low-temperature stress, compared with WT, *DgbZIP2* overexpression lines had significantly enhanced cold tolerance, while antisense suppression lines had significantly reduced cold tolerance (Fig. 4a–c). In addition, the *DgbZIP2* overexpression line reduced the accumulation of reactive oxygen species (REC, MDA, H_2_O_2_, and O^2−^) by regulating *DgPOD* transcript level and POD activity, protecting the stability of cell membranes and improving the tolerance of plants to low-temperature stress (Figs 4f–j and 5g). Therefore, *DgbZIP2* may be an important regulator of cold stress.

Most transcription factors cannot function alone but need to interact with intermediate proteins to initiate transcription. TabZIP15 interacts with the enolase TaENO-b and is involved in the regulation of glycolysis and gluconeogenesis pathways, thereby enhancing the tolerance of wheat to salt stress [45]. DCA1 improves the tolerance of rice to drought and salt stress by regulating the expression of the peroxidase 24 precursor after the interaction of DST and DCA1 [46]. Co-expression of bZIP73^Jap^ and bZIP71 can improve tolerance to low temperature stress during the growth period of rice [47]. OsOBF1 interacts with OsbZIP38/LIP19 and takes an important part in the cold signal transduction pathway [48]. However, the relationship between the two interacting proteins DgbZIP3 and DgbZIP2 and *DgPOD* has not yet been studied in plants. To analyze the interaction of DgbZIP3 and DgbZIP2 on *DgPOD*, EMSA (Fig. 5b), ChIP–qPCR (Fig. 5c), LCI (Fig. 5d), and DLA (Fig. 5e) experiments were performed, and the results showed that DgbZIP2 could not directly regulate the expression of *DgPOD* but enhanced the expression of *DgPOD* through interaction with DgbZIP3. *DgPOD* promoted the activity of POD, regulated the accumulation of ROS, and improved the ability of chrysanthemum to resist low-temperature stress (Supplementary Data Fig. S9). However, whether DgbZIP3 improves cold tolerance is dependent on DgbZIP2, and further investigation would be essential, employing a genetic transformation assay to silence *DgbZIP2* in *DgbZIP3*-overexpressing plants to detect cold tolerance.

In conclusion, the interaction between DgbZIP2 and DgbZIP3 enhanced the expression of *DgPOD*, increased the activity of POD, regulated the balance of ROS, and improved the cold resistance of chrysanthemum.

## Materials and methods

### Experimental materials and low-temperature treatment

In this experiment, WT chrysanthemum seedlings were used as the original plant material. The seedlings were grown on MS medium (200 μM m^−2^ s^−1^) for 30 days (25°C/16 hours light, 22°C/8 hours night, 75% relative humidity), transplanted to peat soil:perlite:vermiculite = 5:1:4 mixed matrix, cultured in a light incubator, and watered once every 3 days. Treatments were imposed when the seedlings grew seven or eight leaves. The normal treatment was 23°C for 32 hours; cold stress treament (4°C) was applied for 0, 3, 6, 12, or 24 h. Samples were stored at −80°C after processing. Phenotypic changes were observed after chilling treatment (−4°C for 8 hours), after which growth was resumed in a constant-temperature incubator (23°C during the day and 21°C at night) for 2 weeks; subsequently, the survival rate of the seedlings was calculated [49].

*Nicotiana benthamiana* seedlings were used as experimental materials. After the tobacco seeds were soaked in water for 24 hours, they were spread on plugs filled with peat soil and cultivated in a light and constant-temperature incubator for 30 days. Water was applied once every 3 days, and the seedlings were transplanted into large pots after they germinated and rooted. When the seedlings grew six or seven leaves, follow-up experiments were carried out.

### RNA extraction and real-time polymerase chain reaction assay

The total RNA in chrysanthemum leaves was extracted using the TIANGEN Polysaccharide Polyphenol Total RNA Extraction Kit, and the complete gold TranScript All-in-One First-Strand cDNA Synthesis SuperMix was used for qPCR. First-strand cDNA was transformed, and then cDNA was added following the method of the PerfectStart™ Green qPCR SuperMix kit. The Bio-Rad CFX96™ detection system was used to perform the real-time PCR (qRT–PCR). *EF1α* was selected as the internal reference gene, and the results were analyzed by the 2^−ΔΔCT^ method. Amplification primers are listed in Supplementary Data Table S1.

### Vector construction

RNA was extracted and reverse-transcribed; primers were designed to amplify the complete open reading frame (ORF) regions of *DgbZIP2* and *DgbZIP3*, which were linked to the pEASY^®^-T5 Zero cloning vector for sequence correction. The corrected ORF regions were ligated into the *pSuper1300-GFP* vector to construct *pSuper1300-DgbZIP2-GFP* and *pSuper1300-DgbZIP3-GFP* overexpression vectors. In the ORF region, a 300 bp fragment without the domain was selected and ligated into the *pCAMBIA2301-GW-RNAi* vector to construct *pCAMBIA2301-DgbZIP2-RNAi* and *pCAMBIA2301-DgbZIP3-RNAi* interference vectors.

### Acquisition of genetically modified chrysanthemum

The *Agrobacterium tumefaciens* GV3101 strain was selected and transformed into recombinant plasmids *pSuper1300-DgbZIP2-GFP*, *pSuper1300-DgbZIP3-GFP*, *pCAMBIA2301-DgbZIP2-RNAi*, and *pCAMBIA2301-DgbZIP3-RNAi* [50], and then transformed into chrysanthemum leaf disks. Different media were used to induce chrysanthemum callus to form seedlings [51]. Antisense suppression lines were designated Ri lines and overexpression lines were designated OE lines.

### Sequence alignment and phylogenetic analysis

The ORF region of the target gene and the BLAST report of the amino acid sequence were analyzed on NCBI, and DNAMAN software was used to analyze the homology of the target gene. MEGA (version 7) software was used to perform systematic evolutionary analysis. The number of branches was expressed as a percentage of the bootstrap value of 1000 sampling repeats, and the scale indicated the branch length.

### Transient expression determination of *Nicotiana benthamiana* leaves

According to previously reported methods [52], with the use of *Agrobacterium*, the constructed fusion protein was injected into tobacco leaves for transient expression. After 36–48 hours, the protein was extracted for subsequent experiments.

### Subcellular localization

After removal of the stop codon from the coding regions of *DgbZIP2* and *DgbZIP3*, they were fused into the *pSuper1300-GFP* vector to form *pSuper1300-DgbZIP2-GFP* and *pSuper1300-DgbZIP3-GFP* recombinant plasmids. *Agrobacterium* GV3101-competent cells were co-transformed with the recombinant plasmid, and the nuclear marker protein (pSuper::NF-YA4-mCherry) was mixed with the transformed bacterial solution, and transformed into tobacco leaves for transient expression. After being subjected to 22°C for 36 hours, the green fluorescence signal was observed using a laser scanning confocal microscope. Empty *pSuper1300-GFP* was used as a control.

### Transcription activity analysis and yeast one-hybrid assay

In order to detect the transcriptional activation activity of the DgbZIP3 transcription factor, a previous method was followed [53], using the following steps. In the vector *pGreen II 0800-LUC*-containing dual luciferase, five *GAL4* sequences and TATA sequences were inserted in front of *LUC* to construct a reporter gene vector, and *REN* was constructed as an internal reference gene in the same vector to form an internal reference reporter. The ORF sequences of *DgbZIP3* and *VP16* were successively connected to the vector containing *GAL4BD* to obtain recombinant plasmids pBD-*DgbZIP3* and pBD-*VP16* to form an effector gene vector. Positive and negative controls were *pBD-VP16* and *pBD-Empty*, respectively.

The above recombinant plasmids were co-transformed with *Agrobacterium* competent GV3101 cells to obtain *Agrobacterium* liquid. The reporter gene bacterial solution was mixed with the effector genes *pBD-DgbZIP3*, *pBD-Empty*, and *pBD-VP16* bacterial solution in equal proportions, and then injected into the tobacco leaves for co-cultivation for 48 hours. The amounts of LUC and REN fluorescence after the reporter gene was expressed in tobacco leaves were determined with a dual-luciferase reporter gene detection kit [54].

The full length and three different deletion coding regions of *DgbZIP3* were fused with the *GAL4* DNA-binding domain (BD) in vecto*r pGBKT7* to form recombinant plasmids *pGBKT7-DgbZIP3* (amino acids 1–336), *pGBKT7-DgbZIP3-1* (amino acids 1–154), *pGBKT7-DgbZIP3-2* (amino acids 155–219), and *pGBKT7-DgbZIP3-3* (amino acids 220–336); *pGBKT7* and *pGAL4* served as negative and positive controls, respectively. The recombinant plasmid was transformed into yeast strain (Y1H), continuously diluted, and inoculated on a double-deficient culture plate (SD/−Ade/−His) cultured with X-α -Gal and 1 mM 3-AT (3-amino-1,2,4-triazole). Growth was observed after 3 days at 30°C.

### Yeast two-hybrid assay

The Y2H test was performed according to the method reported by Liu *et al*. [41]. Full-length *DgbZIP3* and *DgbZIP2* were added to *pGBKT7* and *pGADT7* vectors, respectively. The recombinant plasmids *pGBKT7-DgbZIP3* and *pGADT7-DgbZIP2* were co-transformed into the yeast. Negative controls were *pGBKT7-DgbZIP3* + *pGADT7*, *pGBKT7-Lam* + *pGADT7-DgbZIP2*, and *pGBKT7-Lam* + *pGADT7-T*, while the positive control was *pGBKT7-53* + *pGADT7-T*. The obtained yeast solution was inoculated on double-deficient medium (SD/−Trp/−Leu) plates or quadruple-deficient medium (SD/−Trp/−Leu/−His/−Ade) plates, and the color-developing culture plate was supplemented with X-α-Gal; growth was observed after incubation at 30°C for 3 days.

### Bimolecular fluorescence complementation

The ORF regions of *DgbZIP2* and *DgbZIP3* were ligated into *pCAMBIA1300-YFPn* and *pCAMBIA1300-YFPc* vectors, respectively, to obtain recombinant plasmids *pCAMBIA1300-YFPn-DgbZIP2* and *pCAMBIA1300-YFPc-DgbZIP3*. According to the method of An *et al*. [55], the transformed *Agrobacterium* was mixed and immersed in tobacco leaves, and yellow fluorescent protein (YFP) fluorescence was observed with a laser confocal microscope.

### Vector construction, LCI experiment, and LUC/REN activity analysis

The ORF regions of *DgbZIP2* and *DgbZIP3* were ligated into *pSuper1300* vector, respectively, to obtain *pSuper1300*-*DgbZIP2* and *pSuper1300*-*DgbZIP3* recombinant plasmids. The *pSuper1300* was linked to the *DgPOD* natural promoter sequence and *LUC* reporter gene to obtain the *ProDgPOD-LUC* recombinant plasmid. The internal reference gene *Renilla* luciferase (*REN*) was linked to pSuper1300 to form pSuper1300-REN.

For the LCI experiment, *Agrobacterium* GV3101 was co-transformed with the above-mentioned related recombinant plasmids, and the transformed bacterial solution was injected into tobacco leaves for transient expression. After 36 hours, D-luciferin potassium salt was injected, and fluorescence was observed with a phytoluciferase *in vivo* imaging system. In order to measure the dual luciferases, samples were collected 48 hours after transient expression, the protein was extracted, and the measurement was performed on a microplate reader according to the operation steps of the dual-luciferase reporter gene detection kit.

### Electrophoretic mobility shift assay

The full-length *DgbZIP2* and *DgbZIP3* were cloned into the *pET28a* expression vector to form fusion expression plasmids *pET28a*-*DgbZIP2* and *pET28a*-*DgbZIP3*. The fusion plasmids *pET28a-DgbZIP2* and *pET28a-DgbZIP3* were transformed into chemically competent cells, and the protein was induced with 0.5 mM isopropyl β-d-1 thiogalactopyranoside (IPTG). The protein was extracted from the bacterial solution according to the instructions of the Bacterial Protein Extraction Kit (Sangon Biotech, Shanghai, China). The Ni-NTA Sefinose™ Resin Kit (Sangon Biotech, Shanghai, China) was used to purify the proteins. EMSA analysis was used to select a 54-bp DNA fragment containing a G-box (CACGTC) and A base at the 5′ end in the promoter part of *DgPOD*, and a 6-FAM tag was added at the 5′ end; the competitive probe had the same sequence, and no 6-FAM tag was added to the 5′ end; the mutation probe had the same sequence, all of the bases in G-box (CACGTC) were mutated to A, and the 6-FAM tag was added to the 5′ end. The negative control was His protein. The above probes were all constructed by Sangon Biotech.

### Co-immunoprecipitation

Full-length *DgbZIP2* and *DgbZIP3* were ligated into the *pSuper1300-MYC* and *pSuper1300-GFP* vectors, respectively, to form recombinant plasmids *pSuper1300-DgbZIP2-MYC* and *pSuper1300-DgbZIP3-GFP*. After *pSuper1300-DgbZIP2-MYC*, *pSuper1300-DgbZIP3-GFP*, and empty *pSuper1300-MYC* were transformed into GV3101 *Agrobacterium* competent cells, the *pSuper1300-DgbZIP3-GFP* + *pSuper1300-DgbZIP2-MYC* bacterial liquid was mixed in equal volume and then injected with tobacco leaf dorsal cells for transient expression. The co-expression of *pSuper1300-DgbZIP3-GFP* + *pSuper1300-MYC* was used as a control.

After 48 hours, the protein was extracted and analyzed according to the instructions of the FLAG immunoprecipitation kit. Anti-MYC, Anti-GFP, and horseradish peroxidase-conjugated anti-mouse were used to precipitate antibodies on sodium dodecyl sulfate polyacrylamide gel electrophoresis (SDS–PAGE). The primary antibody was diluted 1:1000 and the secondary antibody was diluted 1:10 000.

### Chromatin immunoprecipitation

In order to study the binding of DgbZIP3 to the *cis*-acting elements in the *DgPOD* promoter region, a 4-week-old *pSuper1300-DgbZIP3-GFP* overexpression line, OE3, was selected. After sampling, it was cross-linked with 1% formaldehyde solution and then immunoprecipitated according to the SimpleChIP^®^ chromatin experimental procedure (magnetic beads) to purify the resulting chromatin preparation. The purified DNA was used for ChIP–qPCR analysis. In the ChIP–qPCR analysis, a fragment containing the *cis*-acting element G-box (CACGTC) in the *DgPOD* promoter region was used. Data processing followed a previous method [41]. The primers used in ChIP–qPCR are detailed in Supplementary Data Table S2.

### Determination of nitroblue tetrazolium and diaminobenzidine staining, and superoxide anion and hydrogen peroxide in chrysanthemum leaves

The accumulation of ROS O^2−^ and H_2_O_2_ in leaves was determined by histochemical staining using NBT and DAB [56]. The contents of O^2−^ and H_2_O_2_ in chrysanthemum leaves were determined following the kit manufacturers’ instructions.

### Determination of physiological indexes of transgenic chrysanthemum under cold stress

After different low-temperature treatments (0, 3, 6, 12, and 24 h at 4°C), transgenic and WT seedlings with consistent growth were sampled. The activities of CAT, POD, APX, and SOD were determined by the method provided with the kits, and determination of the MDA content and REC level were based on previously reported methods [57, 58].

### Statistical analysis

Three biological replicates were used for each experiment. All experimental data were analyzed using SPSS version 25.0, and Duncan’s multiple range test was used for significance analysis (*P* < .05).

## Acknowledgements

We thank Dr Zhizhong Gong for providing *pSuper-1300* and *pSuper-1300-GFP* plasmids, Dr Nan Ma for providing the *pSuper::NF-YA4-mCherry* plasmid, and Dr Jianmin Zhou for providing the *pCAMBIA-35S-cLUC* vector and *pCAMBIA-35S-nLUC* plasmids. This work was supported by the National Natural Science Foundation of China (31971707).

## Author contributions

H.R.B. designed and performed the experiments, conducted data analysis, and wrote the manuscript. X.Q.L., X.L., and B.W. performed the experiments and analyzed the data. Y.C.L., X.H.Y., and Y.C.T performed the experiments; L.Z., F.Z., Y.Z.P., B.B.J., and Y.J. analyzed the data. Q.L. designed the experiment, conceived the project, and supervised the study. All authors have read and approved the final manuscript.

## Data availability

The original sequencing data in the article have been uploaded to NCBI, accession number GSE117262. The datasets generated and analyzed during this study are available from the corresponding author upon reasonable request.

## Conflict of interest

The authors declare no competing interests.

## Supplementary data

Supplementary data is available at *Horticulture Research* online.

## Supplementary Material

Web_Material_uhac105
