# Correction to: DgbZIP3 interacts with DgbZIP2 to increase the expression of *DgPOD* for cold stress tolerance in chrysanthemum

**DOI:** 10.1093/hr/uhaf047

**Published:** 2025-02-24

This is a **correction** to:

Huiru Bai, Xiaoqin Liao, Xin Li, Bei Wang, Yunchen Luo, Xiaohan Yang, Yuchen Tian, Lei Zhang, Fan Zhang, Yuanzhi Pan, Beibei Jiang, Yin Jia, Qinglin Liu, DgbZIP3 interacts with DgbZIP2 to increase the expression of *DgPOD* for cold stress tolerance in chrysanthemum, *Horticulture Research*, Volume 9, 2022, uhac105, 10.1093/hr/uhac105

In the originally published version of this manuscript, the images shown in Figure 4 (b) of the paper, OE2-57, 23 $ ^\circ\pm $ 24h, were not found in the original OE2-57 image database, but were found in the original OE3-68 image database, indicating that this image is the same transgenic strain as the image shown in Figure 3 (b), OE3-68, 4 $ ^\circ $ 24h. Replace the previous images with OE2-157, OE2-57, 23 $ ^\circ\pm $ 2 24h images from the original database.

The author apologize for the wrong conclusion and have corrected Figure 4 (b).



