# Supplementary material for: DgbZIP3 interacts with DgbZIP2 to increase the expression of DgPOD for cold stress tolerance in chrysanthemum
Source: Hortic Res. 2022 May 17;9:uhac105. doi: 10.1093/hr/uhac105 (PMC9271009; doi:10.1093/hr/uhac105)
Supplement: Web_Material_uhac105 [file web_material_uhac105.docx]

**Supplemental Table**

**Supplemental Table 1.** Primers and their sequences in the experiment

| Primer | Sequence(5'-3') |
| --- | --- |
| *DgbZIP3*  *DgbZIP2*  *DgPOD*  *ProDgPOD*    *EF1α* | F：AACCACCATCACATTTACTGCT  R：CCATGGTAAAAGATCAAAATAGC  F：ATGGCGTCACCGGGATCAGATGGAG  R：CTAAAACATATTAATATTGTTCGTTGATGC  F：CCTAAGTCTGTTGGAACAATTTT  R：TAGCAAAATGGATGCATCACA  F：ATACAAGTGTTGCGAAGGTAAGAG  R：CATGTGTCATCATAAAAAGTGGTA  F：TTTTGGTATCTGGTCCTGGAG  R：CCATTCAAGCGACAGACTCA |

**Supplemental Table 2.** ChIP-qPCR primer sequence

| Primer | Sequence(5'-3') |
| --- | --- |
| P1  P2  P3  ACTIN | F：CAACTAATTATTTGCACAGACCG  R：TGTAAGGGGACAGTTTTAGGGA  F：ACGTGTACAAATATCCGTAGCAT  R: TCAATATATATGTGGTATTGAAT  F: TGGGCCAAATTAAGTATCGAA  R: GGCAAGAACAAGCAACACATTA  F：TTTTGGTATCTGGTCCTGGAG  R：CCATTCAAGCGACAGACTCA |

**Supplemental Figures**


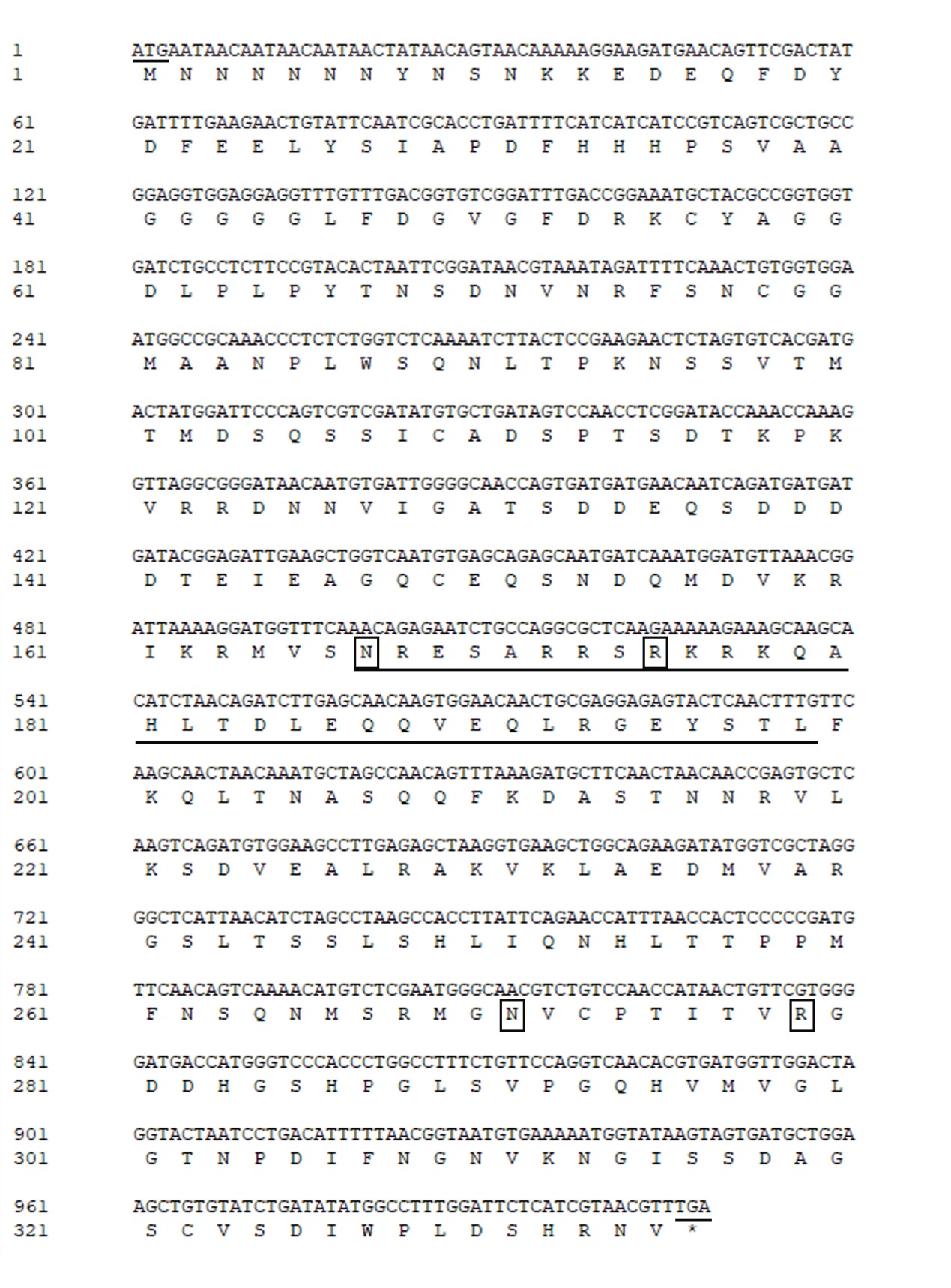


**Figure S1**. The nucleotide sequence and deduced amino acid sequence of *DgbZIP3*. The underlined part is an alkaline area and a leucine zipper area of bZIP.


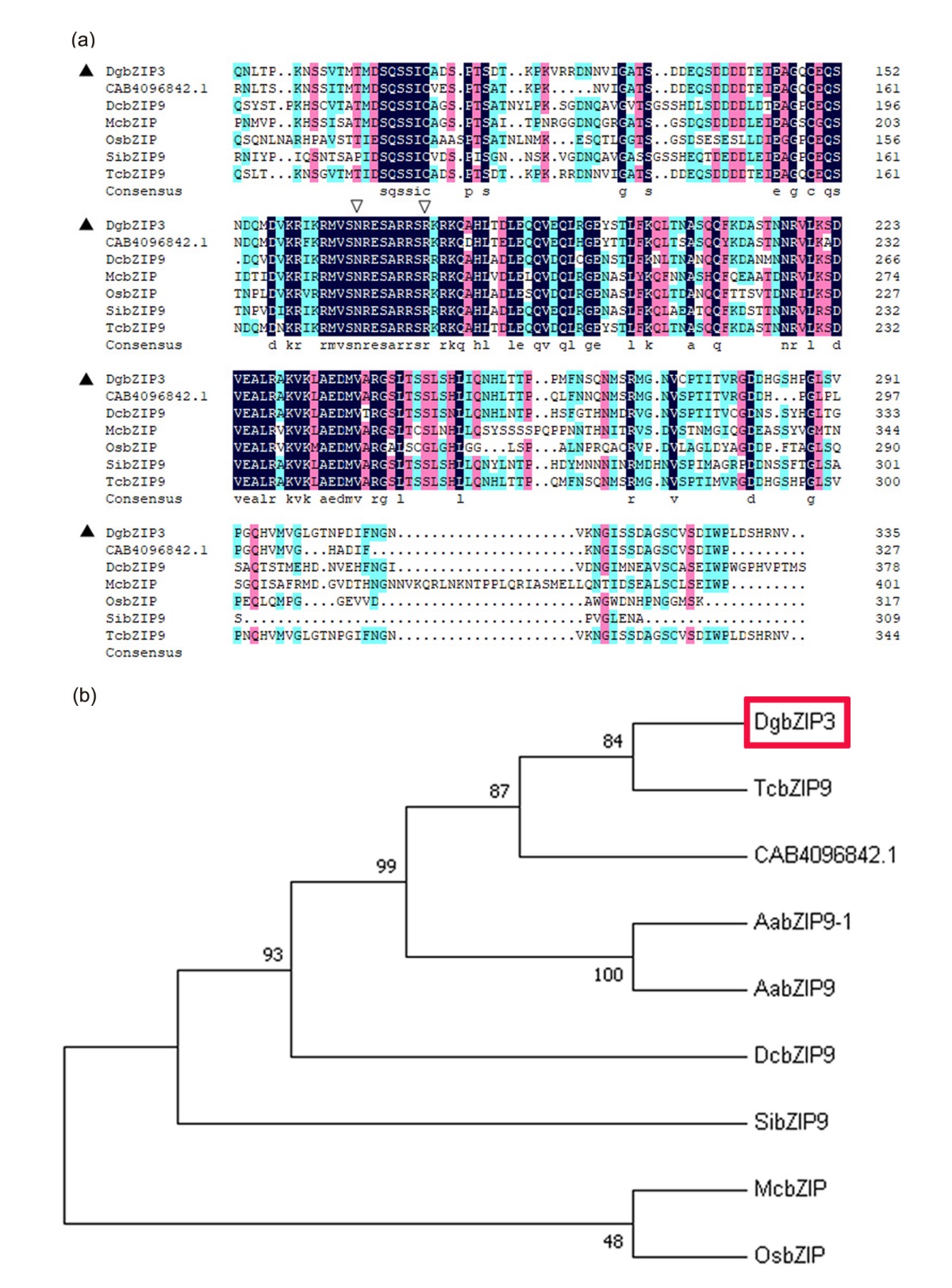


**Figure S2.**  Sequence analysis of DgbZIP3. (a) Multiple alignments of predicted amino acid sequences of DgbZIP3 with other plant bZIP proteins. The relatively conservative structure in the basic region is indicated by an arrow (▽). (b) Phylogenetic tree analysis of the DgbZIP3 sequence with other plant bZIP proteins. DgbZIP3 is highlighted with a red frame. bZIP proteins used in this analysis were as follows: TcbZIP9 (GEY53440.1), Unnamed protein (CAB4096842.1), AabZIP9-1 (QAU20958.1), AabZIP9 (PAW82790.1), DcbZIP9 (XPp_017236158.1), SibZIP9 (XP_011075979.1), McbZIP (OVA19249.1), and OsbZIP (BAF07967.1)


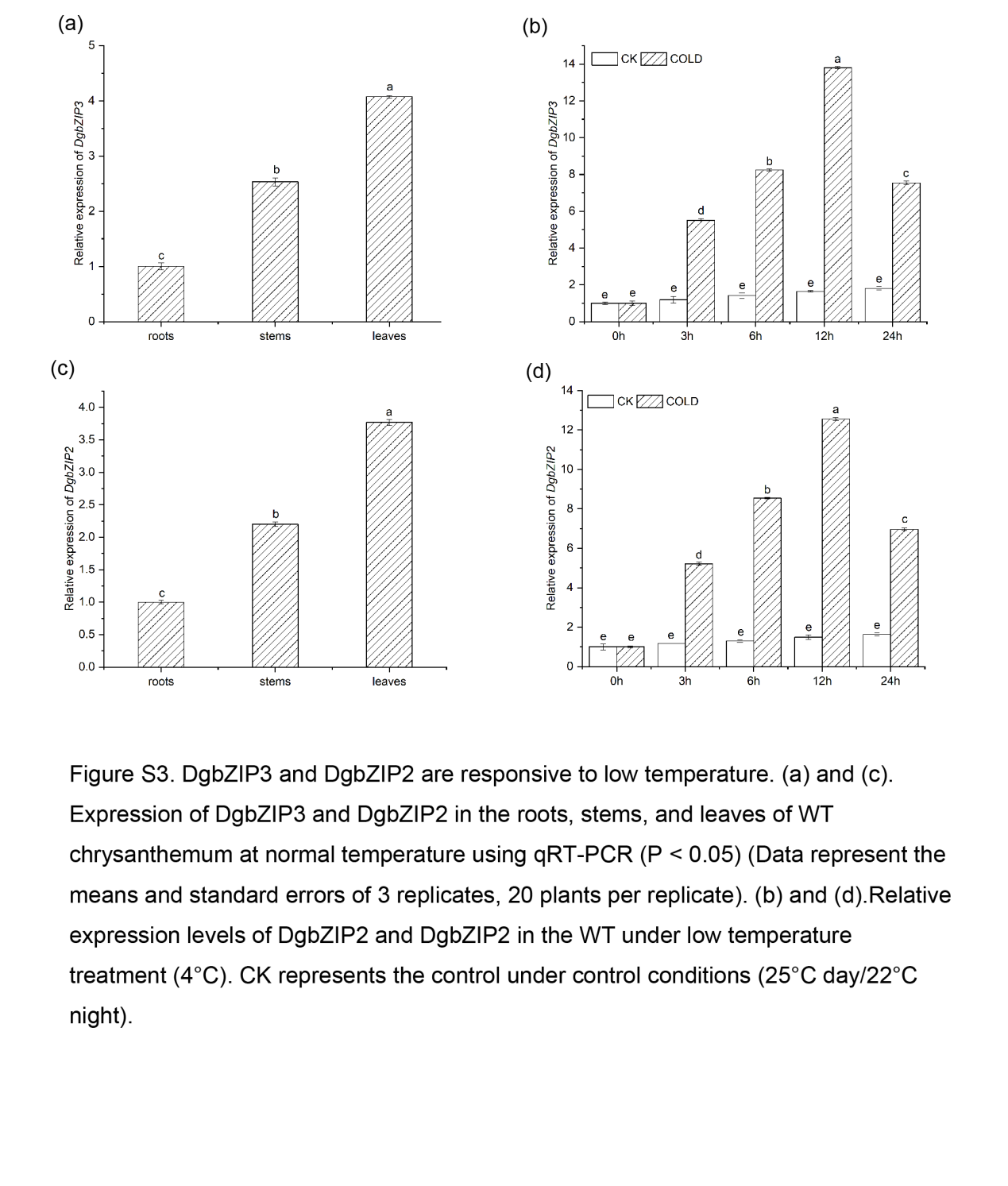


**Figure S3.** *DgbZIP3* is responsive to low temperature. (a) Expression of *DgbZIP3* in the roots, stems, and leaves of WT chrysanthemum at normal temperature using qRT-PCR (P < 0.05) (Data represent the means and standard errors of 3 replicates, 20 plants per replicate). (b) Relative expression levels of *DgbZIP3* in the WT under low-temperature treatment (4°C). CK represents the control under control conditions (25°C day/22°C night).


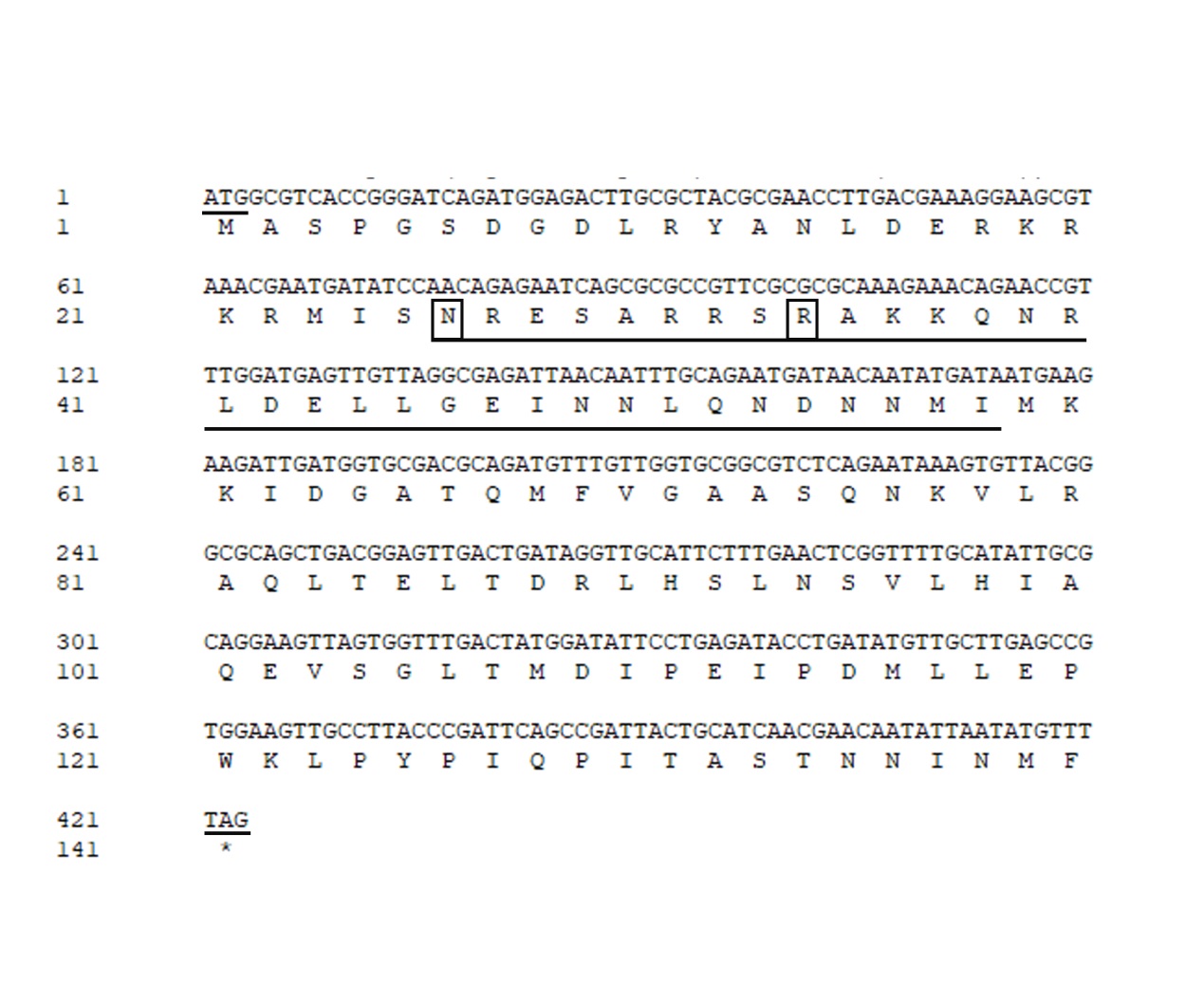


**Figure S4.** The nucleotide sequence and deduced amino acid sequence of *DgbZIP2*. The underlined part is an alkaline area and a leucine zipper area of bZIP.


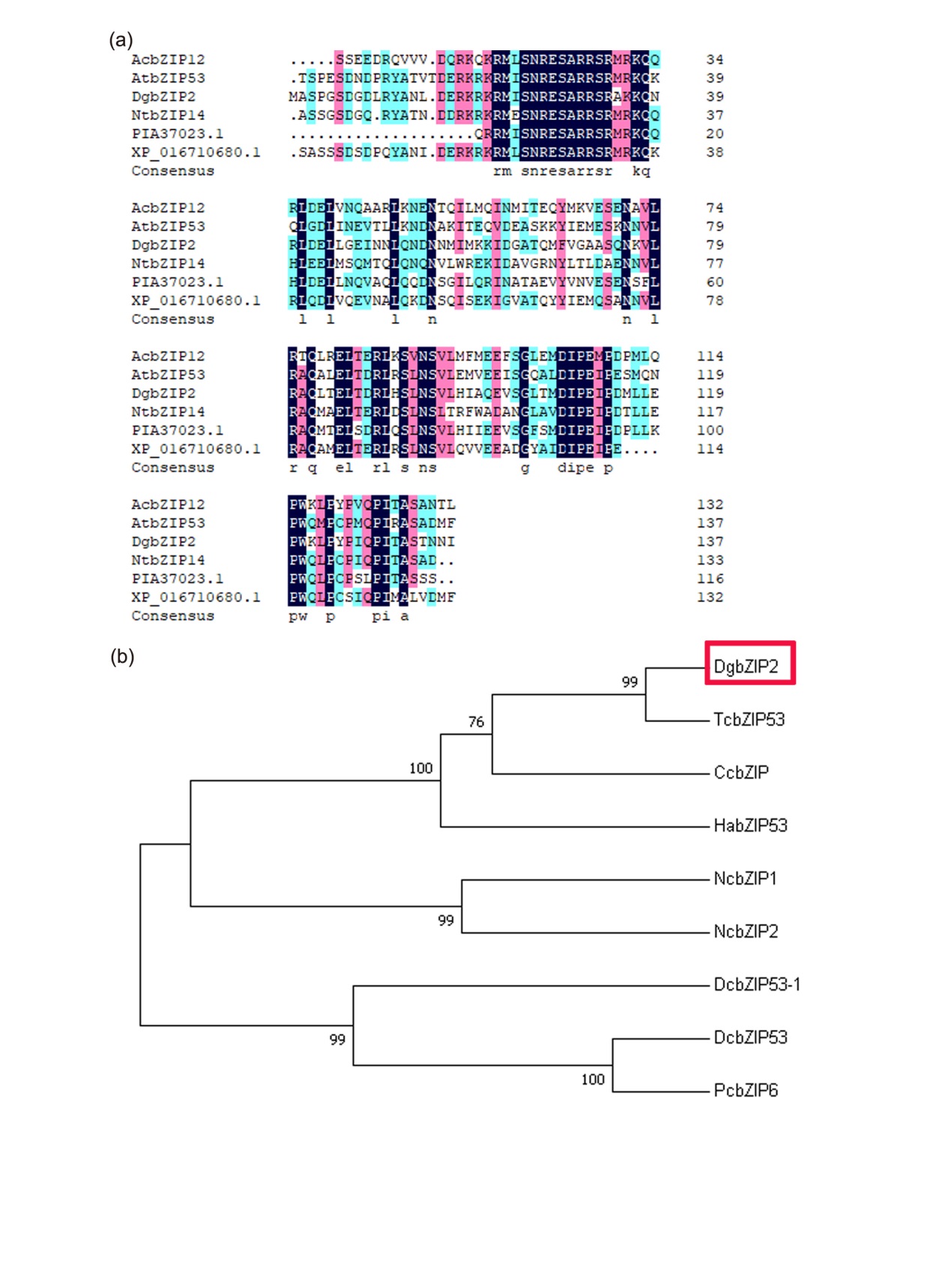


**Figure S5.** Sequence analysis of DgbZIP2. (a) Multiple alignments of predicted amino acid sequences of DgbZIP2 with other plant bZIP proteins. (b) Phylogenetic tree analysis of the DgbZIP2 sequence with other plant bZIP proteins. DgbZIP2 is highlighted with a red frame. bZIP proteins used in this analysis were as follows: NcbZIP1 (kaa8549089.1), NcbZIP2 (kaa8543032.1), CcbZIP (kvi12465.1), HabZIP53 (xp_021969419.1), DcbZIP53 (xp_017230969.1), TcbZIP53 (gev32128.1), PcbZIP6 (cac00657.1), and DcbZIP53-1 (xp_017231721.1)


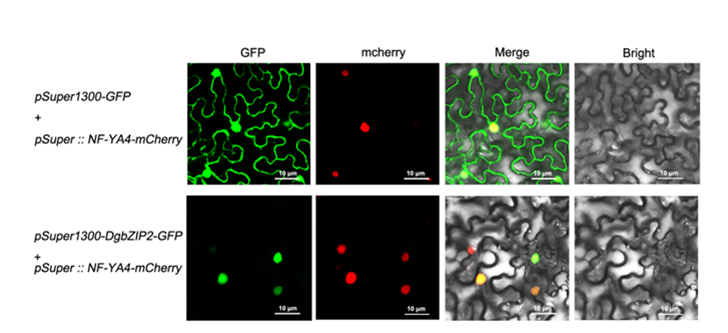


**Figure S6.** Subcellular localization of DgbZIP2 in tobacco leaves. GFP and mCherry were used as negative controls. Scale bars, 10 μm.


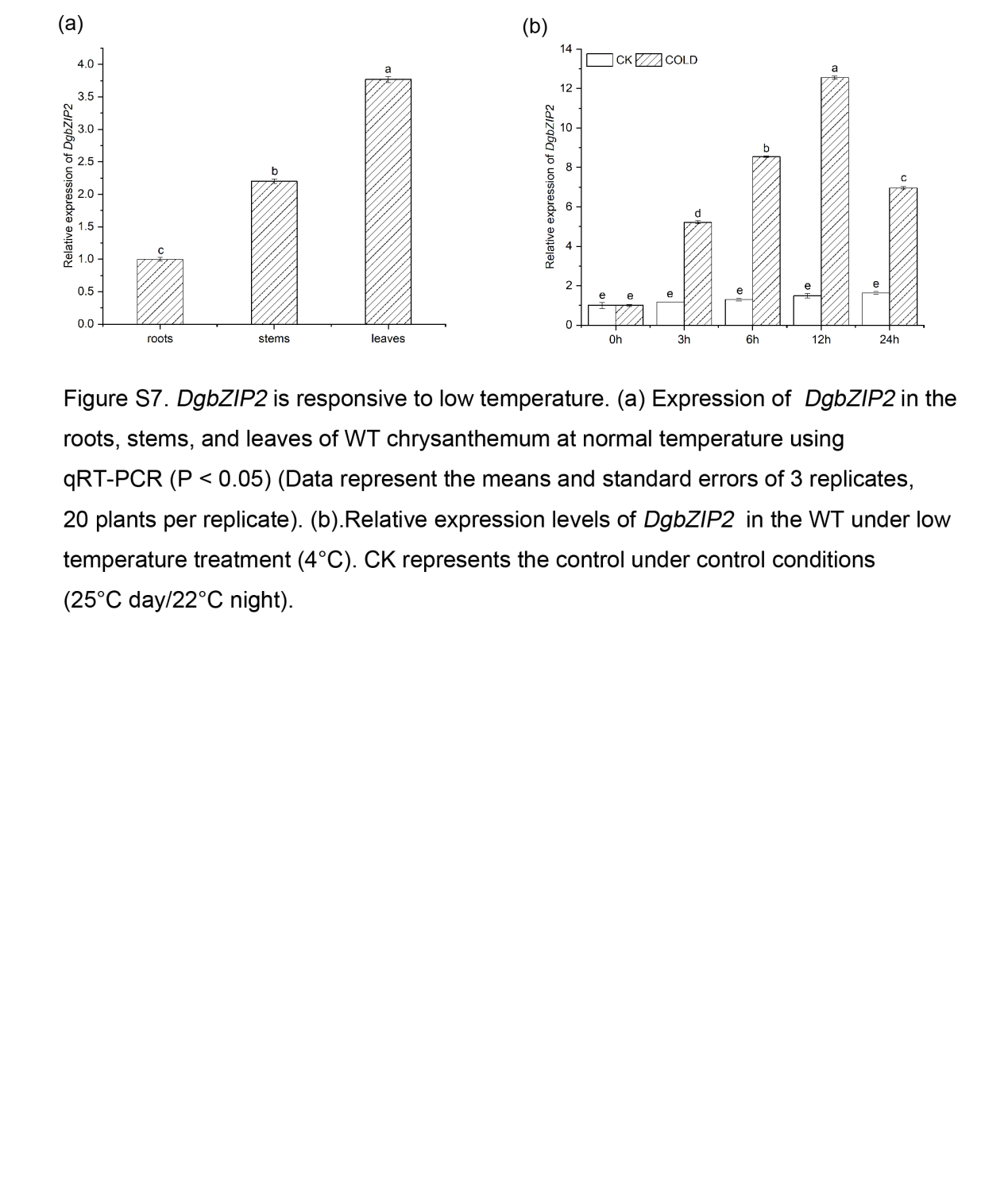


**Figure S7.** *DgbZIP2* is responsive to low temperature. (a) Expression of *DgbZIP2* in the roots, stems, and leaves of WT chrysanthemum at normal temperature using qRT-PCR (P < 0.05) (Data represent the means and standard errors of 3 replicates, 20 plants per replicate). (b) Relative expression levels of *DgbZIP2* in the WT under low-temperature treatment (4°C). CK represents the control under control conditions (25°C day/22°C night).


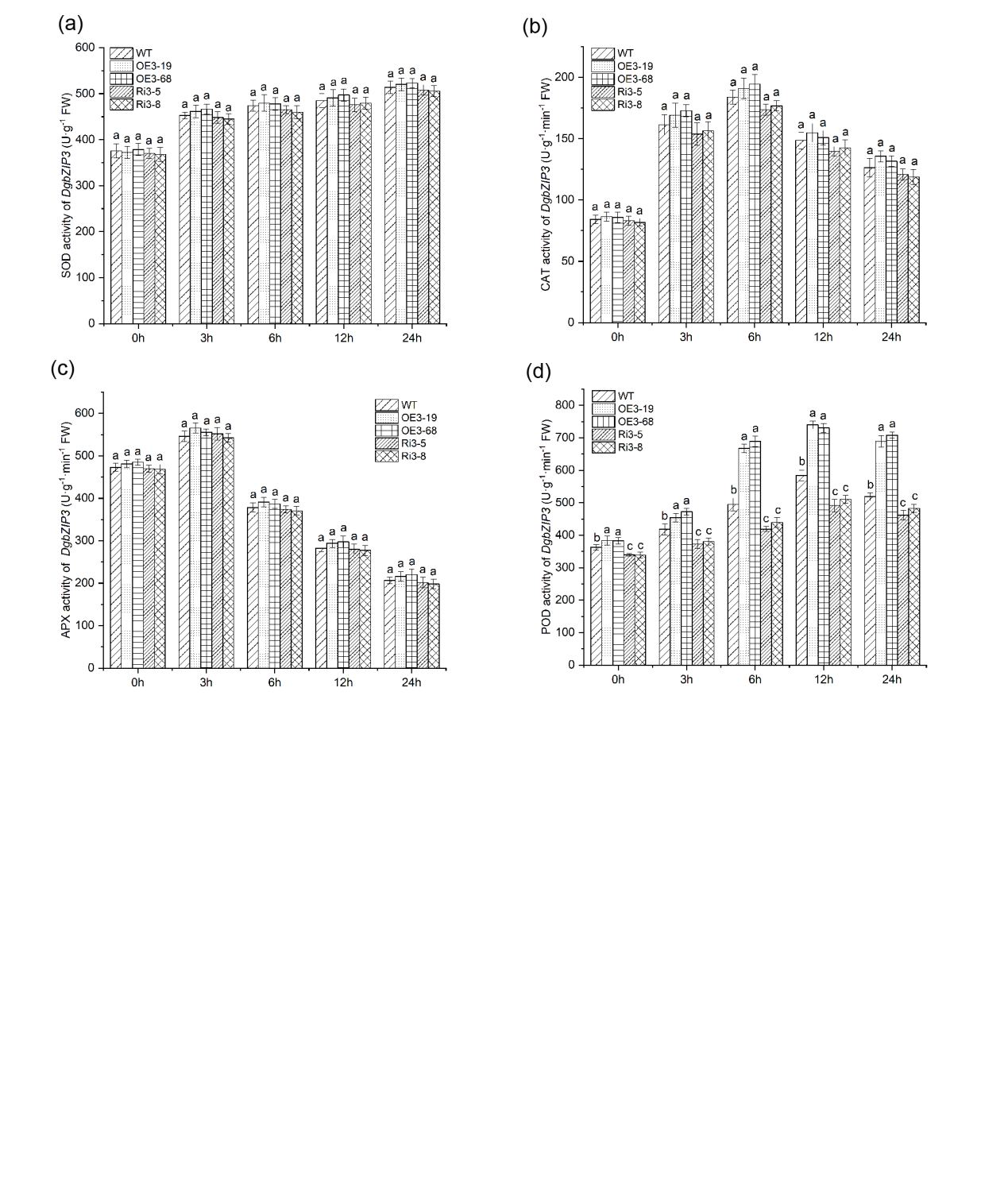


**Figure S8.** Antioxidant enzyme activity in chrysanthemum WT and *DgbZIP3* transgenic lines under low temperature. (a) Superoxide dismutase (SOD) activity. (b) Catalase (CAT) activity. (c) Aseorbateperoxidase (APX) activity. (d) Peroxidase (POD) activity.


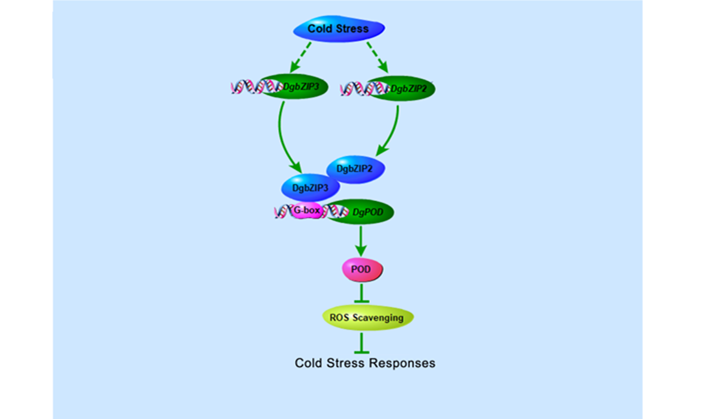


**Figure S9.**  DgbZIP3 and DgbZIP2 interact to adjust the refrigeration stress model. Under cold stress, DgbZIP2 activates the expression of *DgPOD* and the activity of POD through the interaction with DgbZIP3, thereby improving the cold resistance of chrysanthemum.
